# Comparison of tutored group with tutorless group in problem-based mixed learning sessions: a randomized cross-matched study

**DOI:** 10.1186/1472-6920-13-158

**Published:** 2013-12-01

**Authors:** Shogo Hayashi, Koji Tsunekawa, Chikako Inoue, Yoshitaka Fukuzawa

**Affiliations:** 1 Medical Education Center, Aichi Medical University School of Medicine, Nagakute, Aichi 480-1195, Japan; 2 Department of Pharmacology, Aichi Medical University School of Medicine, Nagakute, Aichi, Japan; 3 Aichi Medical University Graduate School of Medicine, Nagakute, Aichi, Japan

**Keywords:** Problem-based learning, Tutorless group, Curriculum, Large class, Learning strategy

## Abstract

**Background:**

Problem-based learning (PBL) involves discussions among students who resolve loosely-structured problems to facilitate learning. In the PBL curriculum, faculty tutors are employed as facilitators for small groups of students. Because of lack of time and staff shortage, the effectiveness of tutorless PBL has been discussed as an alternate option.

**Methods:**

Sessions in which tutored and tutorless PBL groups are mixed were presented by 1^st^-year medical students, who experienced both tutored and tutorless groups alternately in the two sessions of a year. To examine the effectiveness of tutored and tutorless PBL, written examination scores (WES) and self-contentment scores (SCS) were statistically analysed.

**Results:**

WES averages did not significantly differ between the tutored and tutorless groups; however, a significantly greater variation was observed in WES in the tutorless group. SCS averages tended to be higher in the tutored PBL than in tutorless PBL groups.

**Conclusions:**

Students in these tutorless PBL groups performed well in their written examinations, whereas those in the tutored PBL groups, achieved this and reported better self-contentment with their learning experience. Tutorless PBL sessions were considered to be comparable to tutored PBL sessions at least in the early stages.

## Background

In the mid-1960s, problem-based learning (PBL) was adopted as a new approach for medical education at McMaster University, Ontario, Canada. PBL has been described as 'a learning that results from the process of working towards understanding or resolution of a problem’ [1]. PBL not only facilitates the acquisition of knowledge but also that of other generic desirable attributes such as effective communication skills, ability to work in a team (team work), problem-solving skills, self-directed learning ability, ability to share information, appreciate other points of view and identification of personal strengths and weaknesses [2]. Because many of these skills are related to the tutorial process and group dynamics [3], the tutors’ expertise, characteristics and behaviour are believed to influence both the process perspective and learning outcomes [4,5].

In the approach adopted by the McMaster University medical school, a faculty tutor was present during all group activities to monitor, assess and provide immediate input i.e. each group was tutored [6]. Providing a facilitator for PBL can be a problem during times of faculty/staff shortage [6-16]. Therefore, many schools have tried other PBL formats in an attempt to reduce the demands on faculty/staff time and resources; examples of these formats include student-tutored PBL [17] and tutorless PBL [6]. In student-tutored PBL, one student studies the problem in advance and then takes on the role as the tutor of the group instead of the faculty tutor. In tutorless PBL, neither the student tutor nor the faculty tutor is present. There have been many reports suggesting that student-tutored PBL can be just as effective as faculty-tutored PBL with regard to learning outcomes of student-tutored PBL. These sessions can be conducted by senior students [17-19] or also by peer-level students from the same class [3,9,10]. Similarly, a meta-analysis by Leary et al. [20] indicated that student tutors were equally effective when compared with faculty tutors. However, there is limited information on learning outcomes of tutorless PBL [11,14,15].

The aim of this study was to examine the effectiveness of tutorless PBL by comparing learning outcomes between tutorless and tutored groups. Roberts et al. [11] and Kaliyadan et al. [14] reported their experiences with tutorless PBL and concluded that there were no significant differences in learning outcomes between tutored and tutorless PBL. However, in the abovementioned reports, several important differences such as group member characteristics, scenarios or learning materials that were present between the tutored and tutorless PBL conditions were observed. Nicholl and Lou [15] recently reported on tutorless PBL using a model for a large class facilitated by one instructor; they argued that students could achieve the required learning outcomes with tutorless PBL. Moreover, these reports do not only compare learning outcomes between tutored and tutorless PBL because there are many other factors influencing the study and results. To the best of our knowledge, this is the first randomised cross-matched study comparing tutored and tutorless PBL.

## Methods

**Figure 1:**
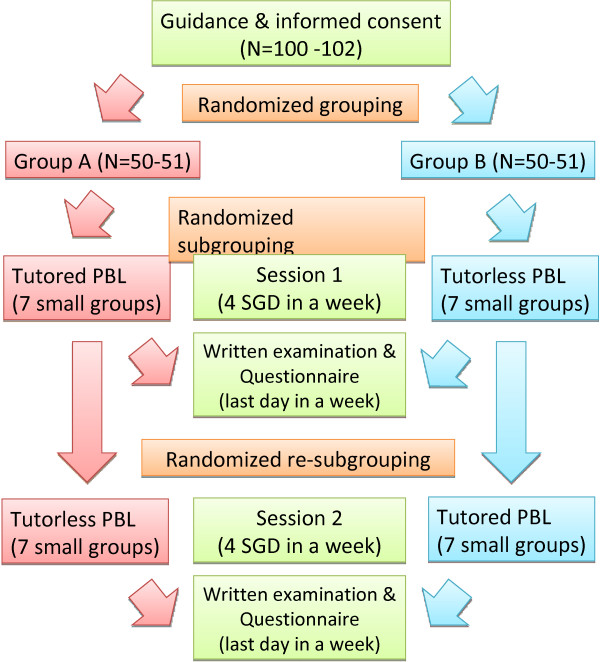
**Chronological list of problem-based learning (PBL) undertaken by students in this study.** Group A in the left row and Group B in the right row. In the middle row, sessions experienced by all students conducted in a large classroom are chronologically displayed.

At Aichi Medical University, the PBL course comprises the following two units: PBL1 for first year medical students and PBL2 for third and-forth year medical students. The goal of PBL1 is introduction and early exposure of clinical medicine to students, and the role of PBL2 is to train students on the art of clinical problem solving. Between 2007 and 2008, as part of the curriculum development of the existing PBL1, we conducted a randomised cross-matched study on the learning outcomes involved in PBL1 (2007, N = 102; 2008, N = 100). The research design is shown in Figure 1. The study was performed in accordance with the provisions of the Declaration of Helsinki. The executive council of the Medical Education Center Aichi Medical University first reviewed the detail protocol, including the future plan of submission. After the review by this committee, the academic affairs department and the faculty council of our school approved this curriculum. The Institutional Review Board exempted the study from review.

**Figure 2:**
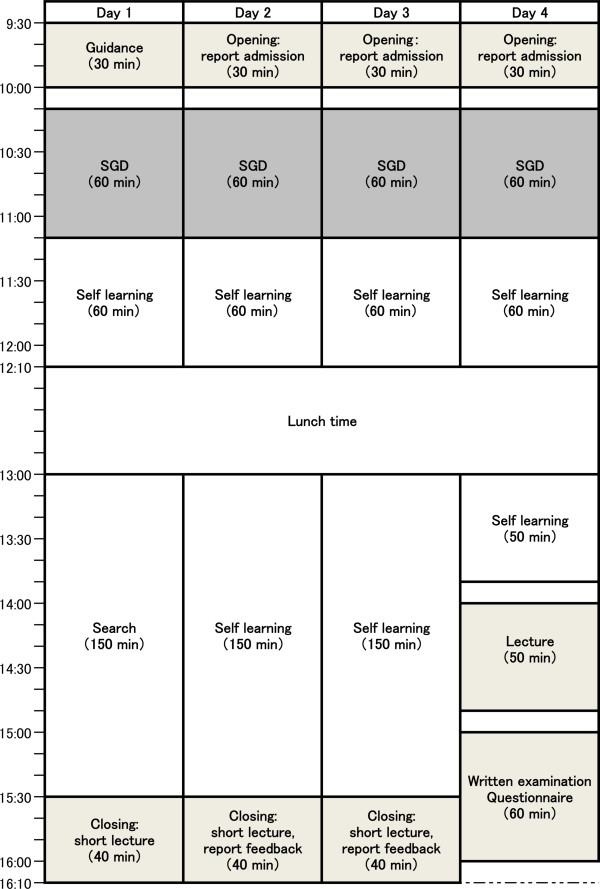
Timetable of each problem-based learning (PBL) session.

These details were explained to all students prior to the start of PBL1, and they were advised that self-evaluation and a questionnaire were voluntary. Students were given the option of not participating in this research, but they all decided to participate. Students were randomly divided into two groups of equal numbers, Group A and Group B. Each group was randomly reorganised into seven small groups comprising seven or eight students for each session. All students attended two sessions in a year; every session covered a four day period (Figure 2). Small group discussions (SGDs) were conducted every day in each session about recurring scenario of chief complaints such as abdominal pain and cough, i.e. four SGDs per session. In group A, SGDs were tutored in the first session and tutorless in the second session. In group B, SGDs were tutorless in the first session and tutored in the second session. Each session was designed such that the schedule for lectures or laboratory practices did not coincide, except for a daily short lecture related to the scenario of each day. Both groups completed a daily report on every SGDs and noted details of any self-learning. On the last day, a written examination (full marks, 100 points) on the contents of each session was conducted. A questionnaire including a 5-tiered self-evaluation on self-contentment and other items shown in Table 1 was simultaneously distributed. An overall evaluation was conducted using a combination of the percentage of attendance and written examination scores (WES). Daily reports, tutor evaluation, self-evaluation and answers to the questionnaire were not included in the summative evaluation.

**Table 1:** Student results on the self-evaluation in each session

	***2007***									
	***Session 1***					***Session 2***				
	**Group A:**		**Group B:**		**p value**	**Group B:**		**Group A:**		**p value**
	**Tutored**		**Tutorless**			**Tutored**		**Tutorless**		
	**(n = 51)**		**(n = 50)**			**(n = 51)**		**(n = 51)**		
	**average**	**SD**	**average**	**SD**		**average**	**SD**	**average**	**SD**	
Self-directed learning	4.19	0.72	3.94	0.71	0.08	4.25	0.74	4.06	0.86	0.22
Activeness	4.13	0.77	4.06	0.71	0.60	4.35	0.72	4.20	0.83	0.31
Scientific basis	3.96	0.77	3.74	0.72	0.14	4.00	0.80	3.89	0.69	0.36
Group dynamics	4.31	0.79	3.88	0.96	0.02	4.57	0.61	4.27	0.72	0.03
Attentiveness	4.20	0.69	4.28	0.67	0.54	4.39	0.72	4.27	0.70	0.40
	***2008***									
	***Session 1***					***Session 2***				
	**Group A:**		**Group B:**		**p value**	**Group B:**		**Group A:**		**p value**
	**Tutored**		**Tutorless**			**Tutored**		**Tutorless**		
	**(n = 46)**		**(n = 45)**			**(n = 45)**		**(n = 46)**		
	**average**	**SD**	**average**	**SD**		**average**	**SD**	**average**	**SD**	
Self-directed Learning	4.41	0.75	4.42	0.70	0.96	4.51	0.59	4.24	0.66	0.037
Activeness	4.35	0.82	4.50	0.71	0.34	4.67	0.52	4.24	0.72	0.001
Scientific basis	4.20	0.78	4.34	0.66	0.33	4.44	0.62	4.14	0.70	0.027
Group dynamics	4.60	0.65	4.56	0.73	0.73	4.56	0.62	4.40	0.78	0.28
Attentiveness	4.50	0.72	4.58	0.57	0.55	4.60	0.50	4.40	0.67	0.19

The average value of WES and self-contentment scores (SCS) for each session was examined with the two-way analysis of variance (ANOVA) and uniformity of examinations was tested with the Tukey–Kramer’s honesty significant difference (HSD) test. The validity of grouping during each year was examined using the unpaired t-test and Bartlett’s test by comparing the total scores of Groups A and B. With regard to WES and self-evaluation results, the average values for the t-test and dispersion of the F-test were compared between the tutored and tutorless PBL groups. The value of p<0.05 (two-tailed) was considered statistically significant. Furthermore, all statistical analyses were conducted using JMP 8.0.1 (SAS institute Inc., Cary, North Carolina, USA) and Prism 6.0b (Graphpad software, Inc., San Diego, CA, USA).

## Results

### Validity of matching and reproducibility of the result

In 2007, the average ± standard deviation of WES in Groups A and B was 131.45 ± 22.32 and 135.86 ± 17.91, respectively. In 2008, WES in Groups A and B was 154.69 ± 29.85 and 135.86 ± 17.91, respectively. No significant difference was observed in the average value or standard deviation between both groups. Therefore, it was concluded that Groups A and B matched in each grade.

While comparing sessions, the average ± standard deviation of WES in 2007 was 64.98 ± 18.74 and 68.68 ± 14.91 for sessions 1 and 2, respectively. In 2008, WES was 76.80 ± 19.76 and 75.25 ± 19.26 for sessions 1 and 2, respectively. The two-way ANOVA recognised a significant effect associated with the year, but no significant effects according to sessions were observed. Furthermore, no significant difference was recognised in the Tukey–Kramer HSD test between sessions 1 and 2 in any year. The average score significantly differed between 2007 and 2008; however, because no difference was observed between the sessions in each year, results of sessions 1 and 2 for each year were considered to be reproducible. Therefore, it was decided that results for each year will be analysed by combining the results of sessions 1 and 2 in groups A and B, respectively, to form the tutored group and those of sessions 1 and 2 in groups B and A, respectively, to form the tutorless group.

### Written examination scores

**Figure 3:**
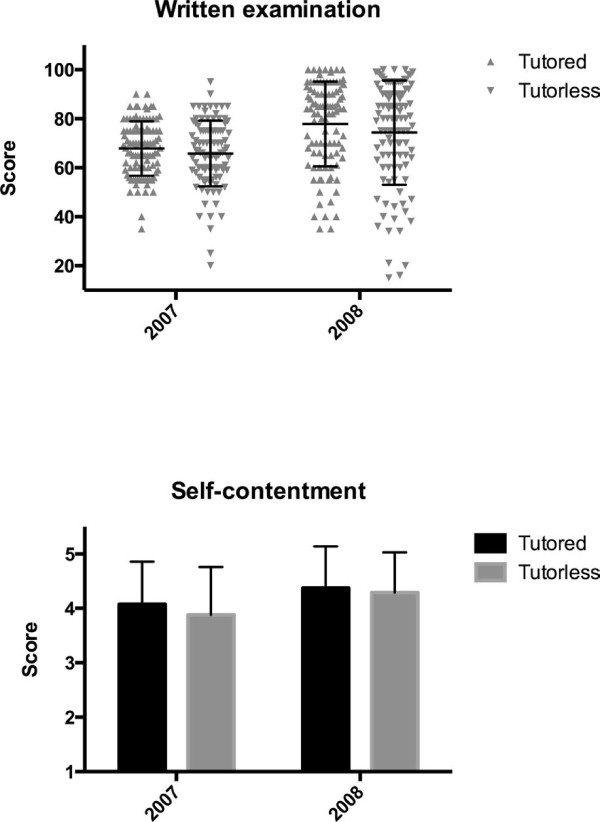
**Students’ performance on the written examination score and self-contentment score during each year.** The error bars represent the standard deviations.

The average ± standard deviation of WES in 2007 was 67.83 ± 11.11 and 65.82 ± 13.42 in the tutored and tutorless groups, respectively. In 2008, WES was 77.85 ± 17.30 and 74.33 ± 21.28 in the tutored and tutorless groups, respectively (Figure 3).

In the tutorless groups, a tendency towards higher average scores during both years was observed, but this difference was not significant (2007, p = 0.25; 2008, p = 0.20). However, in 2008, variances tended to be significantly larger than the average score in the tutorless group (2007, p = 0.058; 2008, p = 0.039).

### Self-contentment scores

In 2007, the average ± standard deviation for SCS was 4.08 ± 0.78 and 3.88 ± 0.88 in the tutored and tutorless groups, respectively. In 2008, SCS was 4.37 ± 0.76 and 4.29 ± 0.74 in the tutored and tutorless groups, respectively (Figure 3). In both years, the average score tended to be lower in the tutorless group but this was not significant (2007, p = 0.092; 2008, p = 0.42). A tendency in the variations from the average scores was inconsistent, and no significant differences were found (2007, p = 0.23; 2008, p = 0.75). No correlation was observed between WES and SCS in both years (2007, r = 0.023, p = 0.74; 2008, r = -0.021 p = 0.76).

### Self-evaluation

Self-evaluation results, excluding SCS, for each session are shown in Table 1. In the tutored group of 2007, there was a tendency for high self-evaluation. In particular, self-evaluation of group dynamics were significantly different. However, inconsistent trends were recognised in 2008. In each student during both years, there was a tendency for high self-evaluation in the tutored PBL session but no significant differences were found. When the results of sessions 1 and 2 for both years were combined, no significant differences were found between the tutored and tutorless groups.

## Discussion

In the present study, a mixed course comprising tutored and tutorless PBL was undertaken by first-year medical students, and the learning outcomes were analysed. The results indicate that students undertaking tutorless PBL were adequately prepared for written examinations, whereas tutored PBL prepares students for written examinations and also increases self-contentment with their learning experience.

Tutorless PBL is an efficient way to reduce demands on faculty time and resources [6,8,11,12,14,15]. However, tutorless PBL may give rise to several problems that may impede learning [21], and according to Duncan-Hewitt [8] these are as follows: (1) students’ emotions can interfere with their willingness to participate and decrease their quality of learning, (2) misapprehensions and weak thinking, group and problem-solving skills can cause students to become engrossed in the problem-solving process, and the problem-solving skills can cause students to concentrate more on the problem-solving process and (3) there is no opportunity to directly examine students’ abilities and skills, which can be systematically improved. Despite these problems, we did not provide any special intervention for students in the tutorless group; however, sufficient guidance was provided to all students in both the tutorless and tutored groups prior to PBL. In addition, following reports regarding SGD and self-learning, we provided formative evaluation and feedback every day. With regard to the fact that WES and SCS did not significantly differ between the tutorless and tutored groups, it appears that these traditional complementary methods may have affected the learning outcomes of the tutorless group.

In contrast, variances in WES scores were considerably greater in the tutorless than tutored groups. Moreover, the average SCS in the tutored group tended to be higher than that in the tutorless group. The daily report also appeared to show that the quality and quantity of SGD and self-learning varied widely in the tutorless group. These results imply that it is unavoidable that tutorless PBL may give rise to some students who do not learn well without a structured process, thereby receiving lower scores. Although the daily short lectures directed the students to study, Lee et al. [22] reported that these were not correlated with perceived self directed learning ability in their case-oriented problem-stimulated mixed PBL curriculum for the 1^st^ and 2^nd^-year medical students. Nicholl and Lou [15] argued that in their tutorless PBL model, it was important to provide as many opportunities as possible for formative assessment in order to monitor and adjust the development of the tutorless groups. We agree with their argument that ongoing and immediate formative assessment is valuable in tutorless PBL. Recent case reports have suggested that the use of pre-set cues, particularly pictures and videos [14] or e-learning resources [11] can help conduct effective tutorless PBL. To improve tutorless PBL, these new learning resources can positively contribute to students’ self-contentment with their learning experience.

A possible reason why WES and SCS did not significantly differ between the tutorless and tutored groups may be because students in the tutorless group communicated with those in the tutored group after every SGD, thereby allowing students in the tutorless group to get some information from those in the tutored group. This helped in decreasing the gap in learning outcomes between both groups. This may also prompt peer-assisted learning for students in both groups. Ross and Cameron [23] argued that peer-assisted learning is an efficient and effective way of preparing medical students for their future role as educators. Although little attention has been paid to the effects of peer-assisted learning in medical schools [24], the positive effects of peer-assisted learning in medical education is gaining notoriety [24,25]. Although there have been no studies comparing tutorless PBL to student-tutored PBL, it appears that the effect of peer-assisted learning in student-tutored PBL is higher than that of tutored and tutorless PBL; however, a mixed course, including tutorless and tutored PBL, has unique characteristics as well as the potential to provide good learning outcomes.

There are two primary limitations to the current study. First, we were unable to develop other methods of measuring the effects of group learning and could measure only self-evaluation of group dynamics and attentiveness. The other types of evaluations such as peer evaluation may be more important in the tutorless group. Second limitation relates to the setting (four SGDs in a week session) and period (pre-clinical students), in which this study was conducted. Usually PBL tutorials are conducted in the first and second sessions with two to three days time for self study. A curriculum in which the SGD frequency is reduced during each session would be required. Moust et al. [26] reported that students in faculty-led PBL performed better than those in peer-facilitated groups during essay examinations designed to assess higher-order cognitive skills. Thus, we completed PBL2 for the third- and forth-year students as a tutored PBL course. More research on the cognitive effects of tutorless PBL for medical students during their clinical years is required.

## Conclusions

Tutorless PBL can potentially produce learning outcomes that are comparable to tutored PBL; however, tutorless PBL is different from faculty/staff-tutored PBL and student-tutored PBL. Tutorless PBL has been used when PBL conducted in large classrooms [6,8,11,12,15]. However, tutorless PBL should not be easily used in the same way as student-tutored PBL because of the difficulty in maintaining faculty tutors or learning rooms. An appropriate and effective curriculum can be administered in every school by combining tutored PBL and student-tutored PBL or tutorless PBL. We encourage the implementation of PBL in schools because this will potentially lead to further developments in the area of PBL.

## Competing interests

The authors declare that they have no competing interests.

## Authors’ contributions

SH conducted all studies, performed statistical analyses and drafted the manuscript. KT and CI helped draft and critically appraise the manuscript. YF was involved in the conceptualisation of the study and participated in its design and coordination. All authors read and approved the final manuscript.

## Pre-publication history

The pre-publication history for this paper can be accessed here:

http://www.biomedcentral.com/1472-6920/13/158/prepub
